# Risk Factors and Dental Caries Incidence in Childhood Cancer Survivors

**DOI:** 10.3390/cancers17061003

**Published:** 2025-03-17

**Authors:** Anna Jodłowska, Danuta Ilczuk-Rypuła

**Affiliations:** Department of Pediatric Dentistry, Medical University of Silesia, 40-055 Katowice, Poland

**Keywords:** chemotherapy in children, adverse effects, dental caries, risk factors, dmf index

## Abstract

Background/Objectives: Dental caries is believed to be one of the most frequent dental long-term adverse effects of anticancer therapy. It may occur due to numerous chemotherapy-dependent oral symptoms or due to the possible neglection of oral care because of parental involvement in the monitoring of other long-term effects of the therapy. This study aimed to determine the incidence of dental caries and the impact of other risk factors in cancer survivors and age-matched controls. Methods: This cross-sectional study was conducted on 40 cancer survivors and 80 peers divided into three age groups. Indices such as dmft/s, DMFT/S, ft/s, FT/S, Plaque Index (PI), and Gingival Index (GI) were calculated to compare the study participants. The sum of dmft and DMFT was used to assess the possible impact of socioeconomic, oral hygiene, and dietary factors. Results: Caries frequency was found to be lower in cancer survivors (92.50%) than in controls (97.50%). No statistically significant differences were found between the study groups within all the caries indices examined. Strong positive correlations with PI and cariogenic diet in the youngest survivors and with PI in middle-aged survivors were observed. Strong negative correlations in middle-aged children were noticed in terms of father’s education in survivors and mother’s education in controls. Conclusions: The study findings suggest that there is no relationship between chemotherapy and dental caries in long-term cancer survivors. Careful dental care still remains a major contributor to maintaining oral health.

## Simple Summary

Dental caries in cancer survivors is believed to maybe occur due to numerous circumstances accompanying anticancer treatment, although they are reported to be transient. The lack of concern for oral health by parents involved in monitoring other adverse effects of anticancer therapy is likely to be of much greater importance. This research aimed to establish a relationship between chemotherapy and caries incidence. The studied survivors and controls represented similar dental caries experience. Moreover, similar correlations have been found in relation to the current socioeconomic and oral hygiene status of the study participants. The authors conclude that there is no relationship between chemotherapy and dental caries in long-term cancer survivors. Careful dental care seems to be a major contributor to maintaining oral health and should always be recommended, especially during anticancer treatment.

## 1. Introduction

There are numerous acute and long-term adverse effects of anticancer treatment involving oral structures. The most frequent long-term dental complications of anticancer therapy are believed to be tooth abnormalities and dental caries [1,2,3,4,5]. While anticancer therapy is known to directly interfere with tooth development, dental caries may occur due to numerous circumstances associated or not associated with cancer treatment. Individuals prone to chemotherapy (CT)- or radiotherapy (RT)-related oral mucosa damage may develop mucositis or candidiasis compromising oral hygiene, and may also experience chewing and swallowing problems [4,5,6,7,8,9,10]. It has been also described that patients reported different taste alterations, nausea, and loss of appetite resulting in changed dietary habits [1,4,7,8,9,10]. Damaged salivary functions may lead to an insufficient supply of minerals and decreased buffering capacity of saliva. Consequently, all these factors may cause changes in the bacterial flora which favor tooth demineralization [7,8,9,10]. The possible neglect of oral care due to the parental involvement in monitoring other long-term adverse effects of the therapy of underlying disease may also be of great importance [10]. A higher prevalence of caries in cancer survivors compared to healthy individuals was reported by some authors, but there are limited studies identifying other influencing factors that seemed to be crucial for caries occurrence [2,9,11,12]. Daily oral hygiene, socioeconomic status, gender, and education are very important to maintain a good condition of the teeth. Numerous clinical and self-reported social, gender, race, and educational inequalities related to oral health have been noticed [2,13,14,15,16,17,18,19,20]. This fact may have a huge impact on the study results in cancer survivors, despite the burden of the general disease. While mucositis is a relatively common treatment-dependent complication in survivors, there is no consistent evidence in the literature that anticancer therapy is related to increased incidence of caries [1,4,7,10,11,15,21,22,23,24,25]. When maintaining proper hygiene, it is possible to keep the oral cavity free from caries. However, it is quite common to believe that due to numerous problems associated with the underlying neoplastic disease and its sequelae, dental needs are often neglected by survivors [1,10].

The aim of this study is to determine the incidence of dental caries and possible impact of important risk factors in cancer survivors and an age-matched control group.

## 2. Materials and Methods

### 2.1. Study Population

This cross-sectional study received approval from the Bioethics Committee of the Medical University of Silesia, Katowice, Poland (KNW/0022/KB1/15/I/13, KNW/0022/KB1/15/II/16, PCN/CBN/0022/KB/112/I/21) and is part of a larger project identifying dental adverse effects of anticancer therapy. Forty cancer survivors aged from 6 years to 17 years and 3 months were recruited into the study from among patients attending the Outpatient Hematology and Oncology Clinic according to the following inclusion criteria: anticancer therapy started before 10 years of age and completed at least two years before the dental examination. The average remission time was 5 years and 5 months (2 years minimum and 11 years and 1 month maximum). All the details about cancer survivors and their oncological treatment are included in Table 1.

Eighty (38 females and 42 males) generally healthy individuals attending the Outpatient Dental Clinic for routine check-ups were qualified to the control group in such a way that there were two age-matched patients for every cancer survivor. Healthy counterparts, aged from 5 years and 8 months to 17 years and 3 months at the time of dental examination, were 4 months younger and 5 months older, maximum. The caregivers of children from experimental and control groups provided their written consent to participate in this study.

### 2.2. Dental Examination

Both groups were examined in a similar way by a qualified specialist in pediatric dentistry. In order to assess the caries status, decay-missing-filled teeth and surfaces for deciduous and permanent teeth were diagnosed, and respective indices, such as dmft, dmfs, DMFT, and DMFS, were established. Caries was diagnosed and recorded at the cavitation level. If both decay and filling were diagnosed in one tooth or in one surface then only decay was revealed in dmft/DMFT and dmfs/DMFS, respectively. A tooth was considered missing if its absence in the oral cavity was the result of carious destruction. To distinguish dental pathology from treatment, f/F scores were calculated separately. Moreover, Löe and Silness Plaque Index (PI) controlling each tooth surface was determined by one examiner using an Oral Health Assessment Form according to WHO indications [26,27]. Additionally, Löe and Silness Gingival Index (GI) was chosen and determined for each tooth surface to check the patient’s periodontal status as a reflection of hygiene level [28]. In the next step, all the participants were divided into three age subgroups in order to properly compare the studied variables: two age groups for mixed dentition (5 y/8 m–9 y/0 m; 9 y/1 m–12 y/0 m) and one for permanent teeth (12 y/1 m–18 y/0 m). In the subsequent survivors’ age groups, there were 18 (45.00%), 15 (37.50%), and 7 (17.50%) patients, respectively. In the control group, the distribution of participants was as follows: 36 (45.00%), 29 (36.25%), and 15 (18.75%), respectively.

### 2.3. Medical Records Analysis and Questionnaire

When it comes to cancer survivors, their medical records were thoroughly analyzed to assess the possible impact of the therapy on caries occurrence. Additionally, information was obtained about oral health status, diet, and the possibility of maintaining proper oral hygiene during anticancer treatment. Moreover, patients from both groups participated in an original survey in order to identify other factors that might contribute to the development of parameters studied. A questionnaire included information concerning the course of pregnancy and childbirth, parental employment and education, number of siblings, tooth brushing frequency, and dietary habits. Parental education was analyzed according to the traditional methods used by national statistical offices. Therefore, 4 subsequent levels were distinguished: elementary taking 8 years of studying (E), 3-year vocational (V), 4–5-year secondary (S), and 5–6-year postsecondary (PS). When one parent had no input into the child’s care, their education level was scored E. Then, correlations between education and dmft + DMFT level were determined separately for each caregiver. Parental employment, the number of siblings, and daily brushing frequency were expressed as a numerical value. Parental occupation was scored 1 if present and 0 if absent during the substantial period of the child’s life. In relation to the above survey point, the sum of the two scores has been established. The zero–one system was adopted for all the other issues included in the questionnaire, making them 1 if the cariogenic diet, complicated pregnancy, and problems with maintaining proper oral hygiene during CT existed. The cariogenic diet was assessed based on several factors: number of snacks per day, type of beverages consumed, consistency of food consumed, and amount of vegetables and grain foods in the daily diet. Due to the fact that the study population included school-aged children, therefore remaining outside their home for a relatively long time, the following qualification criteria for the cariogenic diet were adopted: two or more snacks a day, sweetened beverages, sticky consistency of food, no vegetables, and no grain foods in the daily diet.

### 2.4. Statistical Analysis

To avoid age-dependent false results in evaluating statistical differences between the survivors’ and control groups, both of them were divided into three age ranges. The mean, standard deviation (SD), and median of dmft/DMFT, ft/FT, dmfts/DMFS, Fs/FS, dmft + DMFT, dmfs + DMFS, PI, and GI were established and compared separately for each age range as it is shown in Table 2a–c. The null hypothesis (H0) was adopted that there are no significant differences between the results of the study groups. A non-parametric Mann–Whitney U test was conducted and H0 was rejected if *p* < 0.05. Due to the specificity of the mixed dentition period and the small sample size for some values, the effect size of the Mann–Whitney U test was additionally determined with the use of Glass rank biserial coefficient (rg) and interpreted as small (rg = 0.11 to <0.29), moderate (rg = 0.30 to <0.49), and strong (rg = 0.50 to 1.00).

To analyze the impact of individual risk variables from the study questionnaire included in Table 3a–c and Table 4a–c, a comparison of two or three independent groups was carried out using a non-parametric Mann–Whitney U test or Kruskal–Wallis test, respectively. After a statistically significant difference was found, a post hoc Bonferroni test was used, if applicable. Differences in dental caries incidence were considered significant if *p* < 0.05. Next, a Spearman rank test (rho) was employed for assessing the relationship between dmft + DMFT and particular risk factors, and rho = 0.3–0.5 was accepted as a moderate correlation and rho > 0.5 was considered strong association. The following levels of significance were adopted to the association analysis: *p* < 0.1 was interpreted as a moderate evidence or a trend, and *p* < 0.05 as a strong evidence.

The statistical analysis was carried out with the use of the Statistical Package for the Social Sciences (SPSS), version 29.0 (SPSS Inc., Chicago, IL, USA).

## 3. Results

### 3.1. Caries Frequency

**Figure 1:**
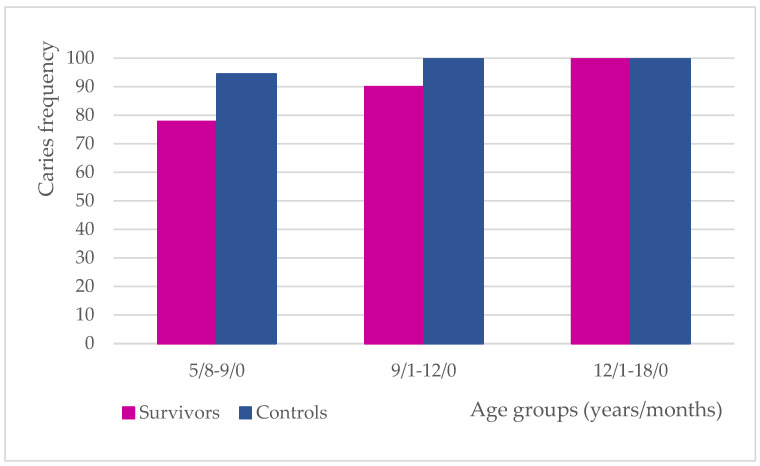
Caries frequency in particular age groups.

The caries frequency was found to be lower in cancer survivors (92.50%) than in the controls (97.50%). Detailed results for each age group have been depicted in Figure 1.

### 3.2. dmft(s)/DMFT(S) Analysis

**Figure 2:**
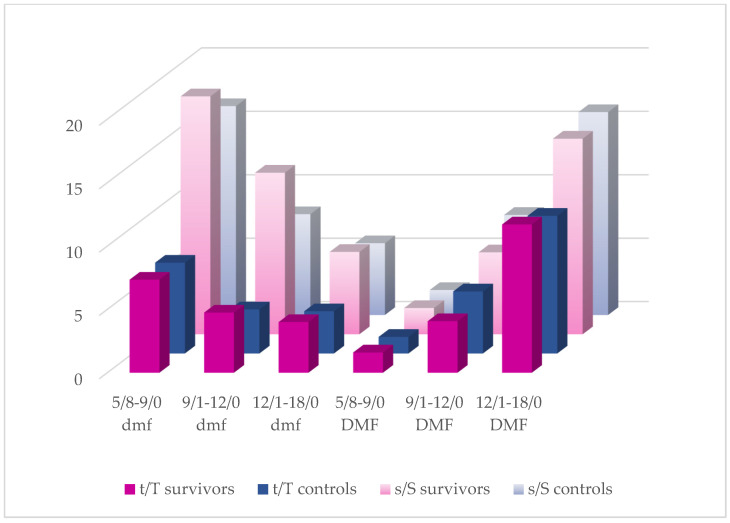
dmft/DMFT and dmfs/DMFS distribution in particular age groups.

As it is shown in Figure 2, almost all the CT recipients presented with higher mean tooth- and surface-related dmf/DMF scores than the controls, except for DMFT and DMFS in the middle-aged group and DMFS in the oldest participants. Among the survivors, lower mean dmft + DMFT in the youngest and middle-aged groups and lower dmfs + DMFS in the middle-aged group were also established. However, within all the caries indices examined, no statistically significant differences were found between the study groups. With regard to treatment factors, mean f/F values have been noticed to be higher in the controls. Except for the FT, fs, and FS scores in the youngest group, the differences were significant (Table 2a–c). The results of the Mann–Whitney U test for primary teeth in the oldest groups were not disclosed due to the small number of patients still using primary teeth.

**Table 2:** (**a**) Caries and oral hygiene expression in groups aged 5/8–9/0. (**b**) Caries and oral hygiene expression in groups aged 9/1–12/0. (**c**) Caries and oral hygiene expression in groups aged 12/1–18/0.

(**a**)								
	**Cancer Survivors** **N = 18**			**Control Group** **N = 36**			**U Mann–Whitney**	
**Variable**	**Mean** **SD**	**Median** **(Range)**	**Median Rank**	**Mean** **SD**	**Median** **(Range)**	**Median Rank**	***p*** **-Value**	**rg**
**dmft**	7.35 ±5.14	8 (0–17)	28.15	7.17 ±4.15	7 (0–17)	26.46	0.709	0.05
**ft**	1.06 ±1.43	1 (0–4)	19.56	2.94 ±2.62	3 (0–8)	30.51	0.013	0.34
**dmfs**	18.76 ±16.21	18 (0–57)	24.53	16.47 ±13.15	13 (0–55)	23.75	0.844	0.03
**fs**	2.47 ±3.63	1 (0–14)	21.80	5.53 ±5.98	4 (0–25)	25.03	0.292	0.15
**DMFT**	1.60 ±1.99	0 (0–6)	28.18	1.31 ±1.59	0.5 (0–4)	26.44	0.703	0.05
**FT**	0.13 ±0.34	0 (0–1)	21.18	0.59 ±1.20	0 (0–4)	29.75	0.055	0.26
**DMFS**	2.07 ±2.86	0 (0–10)	23.97	1.97 ±2.84	0.5 (0–11)	24.02	0.990	<0.01
**FS**	0.40 ±1.02	0 (0–4)	23.50	0.66 ±1.53	0 (0–6)	24.23	0.811	0.03
**dmft + DMFT**	8.28 ±5.17	9 (0–18)	27.78	8.33 ±4.58	8 (0–19)	27.36	0.927	0.01
**dmfs + DMFS**	19.44 ±16.05	19.5 (0–58)	28.22	18.14 ±13.93	15 (0–55)	27.14	0.811	0.03
**PI**	1.058 ±0.60	0.997 (0.229–1.816)	33.31	0.734 ±0.482	0.639 (0.000–1.674)	24.60	0.055	0.26
**GI**	0.381 ±0.33	0.288 (0.000–0.955)	27.31	0.402 ±0.351	0.309 (0.000–1.344)	27.60	0.949	<0.01
**Remission time (months)**	50.83 ±15.37	50 (24–85)		-	-			
	No primary teeth—1patient No permanent teeth—3 patients			No primary teeth—0 patients No permanent teeth—4 patients				
**(b)**								
	**Cancer Survivors** **N = 15**			**Control Group** **N = 29**			**U Mann–Whitney**	
**Variable**	**Mean** **SD**	**Median** **(Range)**	**Median Rank**	**Mean** **SD**	**Median** **(Range)**	**Median Rank**	***p*-Value**	**rg**
**dmft**	4.75 ±4.79	2 (0–12)	16.50	3.48 ±2.14	3 (0–8)	17.16	0.865	0.03
**ft**	0.00 ±0.00	0 (0–0)	10.00	1.16 ±1.54	1 (0–6)	19.24	0.008	0.46
**dmfs**	12.75 ±16.87	3.5 (0–50)	15.13	7.96 ±5.37	8 (0–21)	17.60	0.527	0.11
**fs**	0.00 ±0.00	0 (0–0)	10.00	2.56 ±3.19	2 (0–12)	19.24	0.009	0.46
**DMFT**	4.07 ±3.23	4 (0–14)	20.10	4.90 ±3.33	4 (0–14)	23.74	0.365	0.14
**FT**	0.73 ±1.18	0 (0–4)	15.40	2.17 ±2.00	2 (0–8)	26.17	0.006	0.41
**DMFS**	6.47 ±6.23	4 (0–21)	20.27	7.90 ±7.28	6 (0–31)	23.66	0.405	0.13
**FS**	1.40 ±1.67	0 (0–5)	16.73	3.62 ±3.41	2 (0–12)	25.48	0.028	0.33
**dmft + DMFT**	6.60 ±5.16	5 (0–16)	18.57	7.90 ±3.13	7 (3–14)	24.53	0.142	0.22
**dmfs + DMFS**	13.93 ±14.35	8 (0–56)	19.20	14.83 ±7.90	13 (3–36)	24.21	0.220	0.18
**PI**	1.214 ±0.639	1.087 (0.125–2.163)	23.53	1.126 ± 0.577	1.208 (0–2.196)	21.97	0.701	0.06
**GI**	0.708 ±0.435	0.595 (0–1.667)	22.19	0.472 ± 0.379	0.388 (0–1.602)	25.12	0.477	0.10
**Remission time (months)**	77.00 ±26.90	71 (24–118)		-	-			
	No primary teeth—7 patients No permanent teeth—0 patients			No primary teeth—4 patients No permanent teeth—0 patients				
(**c**)								
	**Cancer Survivors** **N = 7**			**Control Group** **N = 15**			**U Mann–Whitney**	
**Variable**	**Mean** **SD**	**Median** **(Range)**	**Median Rank**	**Mean** **SD**	**Median** **(Range)**	**Median Rank**	***p*** **-Value**	**rg**
**dmft**	4.00 ^1^ ±1.00	4 (3–5)	-	3.33 ±0.94	4 (2–4)	-	-	-
**ft**	0.50 ^1^ ±0.50	0.5 (0–1)	-	1.00 ±0.82	1 (0–2)	-	-	-
**dmfs**	6.50 ^1^ ±2.50	6.5 (4–9)	-	5.67 ±2.05	6 (3–8)	-	-	-
**fs**	0.50 ^1^ ±0.50	0.5 (0–1)	-	1.33 ±0.94	2 (0–2)	-	-	-
**DMFT**	11.71 ±5.39	13 (4–19)	11.93	10.87 ±4.72	13 (2–20)	11.30	0.831	0.05
**FT**	1.71 ±2.31	1 (0–7)	7.57	4.13 ±3.10	4 (1–12)	13.33	0.050	0.42
**DMFS**	15.43 ±8.31	16 (4–29)	11.14	16.00 ±7.55	16 (3–34)	11.67	0.860	0.04
**FS**	2.57 ±2.56	1 (0–7)	7.71	6.33 ±4.95	4 (1–18)	13.27	0.060	0.40
**dmft + DMFT**	12.86 ±3.87	13 (7–19)	12.29	11.53 ±4.05	13 (2–20)	11.13	0.694	0.08
**dmfs + DMFS**	17.29 ±6.50	16 (8–29)	11.00	17.13 ±6.68	17 (3–34)	11.73	0.805	0.05
**PI**	1.193 ±0.645	1.509 (0.228–1.929)	14.14	0.860 ±0.388	0.798 (0.313–1.556)	10.27	0.192	0.28
**GI**	0.572 ±0.305	0.554 (0.141–1.130)	13.43	0.423 ±0.304	0.339 (0.028–1.020)	10.60	0.341	0.20
**Remission time (months)**	75.14 ±37.29	74 (24–133)		-	-			
	No primary teeth—5 patients No permanent teeth—0 patients			No primary teeth—12 patients No permanent teeth—0 patients				

^1^ Statistically insufficient number of patients with primary teeth. rg Glass rank coefficient.

### 3.3. Comparison of Mean PI and GI Scores

As described in Table 2a–c, the survivors had higher mean PI than the controls, but, except for the age group of 5/8–9/0, the differences were not significant. When it comes to the GI value, only the youngest survivors showed a slightly lower mean score compared to the control group.

### 3.4. dmft + DMFT and Different Risk Factors

In response to the survey questions, the authors obtained information about the study population (Table 3a–c and Table 4a–c). In the group of survivors, eight mothers declared complicated pregnancy and birth problems as follows: cholestasis of pregnancy (1), gestational diabetes (1), high-risk pregnancy (1), bacterial infection (1), preterm birth (PTB) (1), and low birth weight (LBW) (3). In the control group, the following problems were reported: viral and bacterial infection (2), preeclampsia (1), gestational diabetes (1), gestational hypertension (4), high-risk pregnancy (1), PTB (3), and LBW (3).

Based on the information obtained from the oral hygiene examination as well as from the survey, the relationships between caries incidence and chosen risk factors were established. Children with the cariogenic diet presented with significantly higher dmft + DMFT in both groups aged 5/8–9/0 (*p* = 0.006; *p* = 0.038). A significantly higher caries incidence was also noted in middle-aged controls whose mothers had elementary education relatively to those with postsecondary school completed (*p* = 0.011). Some differences in dmft + DMFT levels with regard to PI, parental employment, and the number of siblings were also noticed; however, they were of lower significance. No significant differences in caries incidence in relation to all the questionnaire variables were found in the oldest study groups (Table 3a–c and Table 4a–c).

The study relationships analysis showed strong positive correlations with oral hygiene (PI) and the cariogenic diet in the youngest survivors (rho = 0.61, *p* = 0.008; rho = 0.67, *p* = 0.002) and with PI in middle-aged survivors (rho = 0.54, *p* = 0.038). Moderate negative correlations in middle-aged children were noticed in terms of parental education in general (rho (survivors) = −0.44, *p* = 0.103; rho (controls) = −0.47, *p* = 0.011; results not included), father’s education in survivors (rho = −0.59, *p* = 0.022), and mother’s education in controls (rho = −0.55, *p* = 0.002). We also observed a higher number of questionnaire variables strongly or moderately correlated with dmft + DMFT level in cancer survivors compared to the controls (Table 3a–c and Table 4a–c).

**Table 3:** (**a**) dmft + DMFT and different risk factors in cancer survivors’ group in the age range 5/8–9/0. (**b**) dmft + DMFT and different risk factors in cancer survivors’ group in the age range 9/1–12/0. (**c**) dmft + DMFT and different risk factors in cancer survivors’ group in the age range 12/1–18/0.

(**a**)							
**Variable**	**Categories** **N (%)** **(18 Survivors)**				**Spearman Correlation**		**Kruskall–Wallis/** **Mann–Whitney**
**Age Range 5/8–9/0**					**rho**	***p*** **-Value**	***p*** **-Value**
Oral hygiene	**Good**	**Fair**		**Poor**			
	10 (55.56)	8 (44.44)		0 (0.00)	0.61	0.008	0.068 ^b^
Parental education	**E/V**	**S**		**PS**			
Women	1 (5.55)	7 (38.89)		10 (55.56)	0.11	0.652	0.892 ^a^
Men	2 (11.11)	11 (61.11)		5 (27.78)	−0.19	0.452	0.488 ^a^
Parental employment	**0**	**1**		**2**			
	0 (0.00)	9 (50.00)		9 (50.00)	0.18	0.469	0.452 ^b^
Number of siblings	**0**	**1**		**≥2**			
	5 (27.78)	11 (61.11)		2 (11.11)	0.48	0.044	0.138 ^a^
Brushing frequency	**0**	**1**		**≥2**			
	1 (5.55)	3 (16.67)		14 (77.78)	−0.45	0.063	0.176 ^a^
Diet	**0**		1 *				
	11 (61.11)		7 (38.89)		0.67	0.002	0.006 ^b^
Pregnancy	**0**		**1**				
	13 (72.22)		5 (27.28)		0.06	0.813	0.805 ^b^
Oral hygiene problems during CT	0		1				
	12 (66.67)		6 (33.33)		0.26	0.294	0.280 ^b^
(**b**)							
**Variable**	**Categories** **N (%)** **(15 Survivors)**				**Spearman Correlation**		**Kruskall–Wallis/** **Mann–Whitney**
**Age Range 9/1–12/0**					**rho**	***p*** **-Value**	***p*** **-Value**
Oral hygiene	**Good**	**Fair**		**Poor**			
	5 (3.33)	10 (66.67)		0 (0.00)	0.54	0.038	0.222 ^a^
Parental education	**E/V**	**S**		**PS**			
Women	4 (26.67)	4 (26.67)		7 (46.66)	−0.48	0.073	0.140 ^a^
Men	4 (26.67)	6 (40.00)		5 (33.33)	−0.59	0.022	0.062 ^a^
Parental employment	**0**	**1**		**2**			
	0 (0.00)	6 (40.00)		9 (60.00)	−0.25	0.363	0.344 ^b^
Number of siblings	**0**	**1**		**≥2**			
	1 (6.67)	9 (60.00)		5 (33.33)	0.17	0.550	0.377 ^a^
Brushing frequency	**0**	**1**		**≥2**			
	0 (0.00)	1 (6.67)		14 (93.33)	−0.31	0.260	0.245 ^a^
Diet	**0**		**1**				
	13 (86.67)		2 (13.33)		0.25	0.368	0.349 ^b^
Pregnancy	**0**		**1**				
	12 (80.00)		3 (20.00)		0.00	1.000	1.000 ^b^
Oral hygiene problems during CT	**0**		**1**				
	11 (73.33)		4 (26.67)		−0.21	0.452	0.432 ^b^
(**c**)							
**Variable**	**Categories** **N (%)** **(7 Survivors)**				**Spearman Correlation**		**Kruskall–Wallis/** **Mann–Whitney**
**Age Range 12/1–18/0**					**rho**	***p*** **-Value**	***p*** **-Value**
Oral hygiene	**Good**	**Fair**		**Poor**			
	2 (28.57)	5 (71.43)		0 (0.00)	0.20	0.670	0.241 ^b^
Parental education	**E/V**	**S**		**PS**			
Women	1 (14.29)	4 (57.14)		2 (28.57)	−0.26	0.571	0.600 ^a^
Men	3 (42.86)	3 (42.86)		1 (14.28)	−0.43	0.338	0.559 ^a^
Parental employment	**0**	**1**		**2**			
	0 (0.00)	5 (71.43)		2 (28.57)	0.32	0.485	0.434 ^b^
Number of siblings	**0**	**1**		**≥2**			
	3 (42.86)	3 (42.86)		1 (14.28)	0.35	0.441	0.312 ^a^
Brushing frequency	**0**	**1**		**≥2**			
	2 (28.57)	2 (28.57)		3 (42.86)	−0.19	0.682	0.506 ^a^
Diet	**0**		**1**				
	2 (28.57)		5 (71.43)		−0.16	0.733	0.696 ^b^
Pregnancy	**0**		**1**				
	6 (85.72)		1 (14.28)		−0.62	0.139	0.130 ^b^
Oral hygiene problems during CT	**0**		**1**				
	5 (71.43)		2 (28.57)		0.16	0,733	0.696 ^b^

Oral hygiene (PI): Good (0.0–0.999); Fair (1.0–1.999); and Poor (2.0–3.0). Parental (caregivers’) education: E/V (elementary/vocational); S (secondary); and PS (postsecondary). Parental (caregivers’) employment: 0 (lack of employment); 1 (1 parent employed); and 2 (2 parents employed). Diet: 0 (proper) and 1 (carious). Pregnancy: 0 (not complicated) and 1 (complicated). Oral hygiene problems during CT: 0 (no) and 1 (yes). ^a^—Kruskall–Wallis test performed; ^b^—Mann–Whitney test performed. * Category with higher mean dmft + DMFT if statistically significant difference was established.

**Table 4:** (**a**) dmft + DMFT and different risk factors in control group in the age range 5/8–9/0. (**b**) dmft + DMFT and different risk factors in control group in the age range 9/1–12/0. (**c**) dmft + DMFT and different risk factors in control group in the age range 12/1–18/0.

(**a**)								
**Variable**	**Categories** **N (%)** **(36 Controls)**				**Spearman Correlation**			**Kruskall–Wallis/** **Mann–Whitney**
**Age Range 5/8–9/0**					**rho**		***p*** **-Value**	***p*** **-Value**
Oral hygiene	**Good**	**Fair**		**Poor**				
	23 (63.89)	13 (36.11)		0 (0.00)	0.21		0.229	0.740 ^b^
Parental education	**E/V**	**S**		**PS**				
Women	8 (22.22)	22 (61.11)		6 (16.67)	0.12		0.505	0.794 ^a^
Men	16 (44.44)	16 (44.44)		4 (11.12)	−0.09		0.610	0.874 ^a^
Parental employment	**0**	**1**		**2**				
	2 (5.55)	15 (41.67)		19 (52.78)	−0.26		0.123	0.167 ^a^
Number of siblings	**0**	**1**		**≥2**				
	8 (22.22)	17 (47.22)		11 (30.56)	0.27		0.113	0.067 ^a^
Brushing frequency	**0**	**1**		**≥2**				
	7 (19.44)	2 (5.56)		27 (75.00)	0.13		0.448	0.739 ^a^
Diet	**0**		1 *					
	17 (47.22)		19 (52.78)		0.35		0.036	0.038 ^b^
Pregnancy	**0**		**1**					
	29 (80.56)		7 (19.44)		0.05		0.753	0.748 ^b^
(**b**)								
**Variable**	**Categories** **N (%)** **(29 Controls)**				**Spearman Correlation**			**Kruskall–Wallis/** **Mann–Whitney**
**Age Range 9/1–12/0**					**rho**		***p*** **-Value**	***p*** **-Value**
Oral hygiene	**Good**	**Fair**		**Poor**				
	11 (37.93)	15 (51.72)		3 (10.35)	0.12		0.549	0.186 ^a^
Parental education	E/V*	**S**		**PS**				
Women	6 (20.69)*	16 (55.17)		7 (24.14)	−0.55		0.002	0.014 ^a^
Men	14 (48.28)	13 (44.82)		2 (6.90)	−0.26		0.167	0.335 ^a^
Parental employment	**0**	**1**		**2**				
	2 (6.90)	9 (31.03)		18 (62.07)	−0.35		0.061	0.070 ^a^
Number of siblings	**0**	**1**		**≥2**				
	2 (6.90)	16 (55.17)		11 (37.93)	0.41		0.028	0.061 ^a^
Brushing frequency	**0**	**1**		**≥2**				
	6 (20.69)	5 (17.24)		18 (62.07)	−0.26		0.172	0.356 ^a^
Diet	**0**		**1**					
	16 (55.17)		13 (44.83)		−0.04		0.846	0.842 ^b^
Pregnancy	**0**		**1**					
	22 (75.86)		7 (24.14)		−0.10		0.616	0.607 ^b^
(**c**)								
**Variable**	**Categories** **N (%)** **(15 Controls)**					**Spearman Correlation**		**Kruskall–Wallis/** **Mann–Whitney**
**Age Range 12/1–18/0**						**rho**	***p*** **-Value**	***p*** **-Value**
Oral hygiene	**Good**	**Fair**		**Poor**				
	9 (60.00)	6 (40.00)		0 (0.00)		0.41	0.129	0.188 ^b^
Parental education	**E/V**	**S**		**PS**				
Women	3 (20.00)	7 (46.67)		5 (33.33)		−0.16	0.582	0.722 ^a^
Men	7 (46.67)	5 (33.33)		3 (20.00)		−0.06	0.846	0.972 ^a^
Parental employment	**0**	**1**		**2**				
	1 (6.67)	5 (33.33)		9 (60.00)		−0.36	0.193	0.386 ^a^
Number of siblings	**0**	**1**		**≥2**				
	1 (6.67)	8 (53.33)		6 (40.00)		0.36	0.191	0.215 ^a^
Brushing frequency	**0**	**1**		**≥2**				
	2 (13.33)	3 (20.00)		10 (66.67)		−0.16	0.570	0.829 ^a^
Diet	**0**	**1**						
	6 (40.00)	9 (60.00)				0.29	0.299	0.282 ^b^
Pregnancy	**0**	**1**						
	14 (93.33)	1 (6.67)				−0.44	0.101	0.100 ^b^

* According to post hoc Bonferroni test children of mothers with elementary education had statistically significantly higher mean dmft + DMFT compared to children whose mothers presented with PS education level (*p* = 0.011). ^a^—Kruskall–Wallis test performed; ^b^—Mann–Whitney test performed.

### 3.5. Gender Distribution in This Study

**Figure 3:**
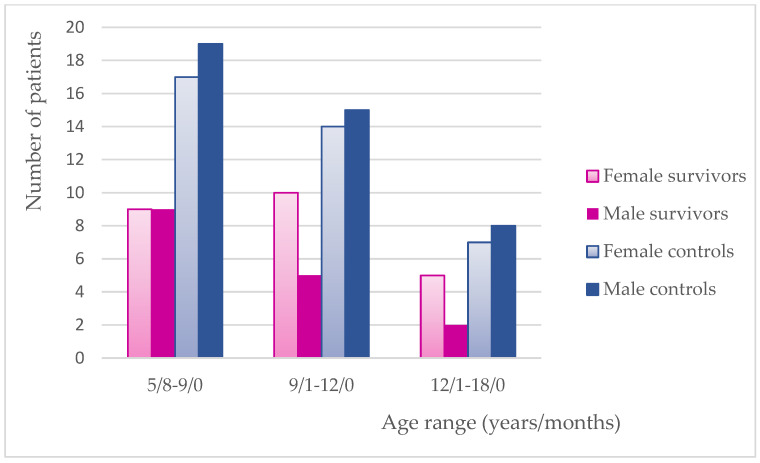
Gender distribution in particular age groups.

**Figure 4:**
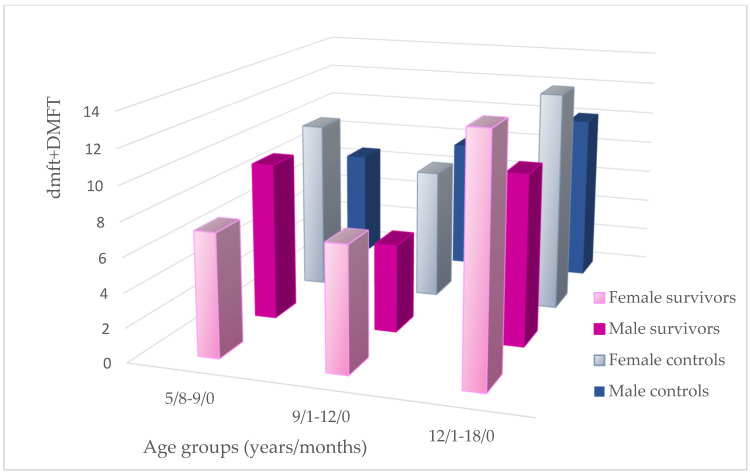
Gender distribution of mean dmft + DMFT in particular age groups.

Gender distribution has been performed in the studied groups for the need of the relationship analysis. The two youngest study groups and the remaining controls had an almost equal gender representation. In terms of dmft + DMFT values, females presented with higher mean scores except for the youngest survivors and middle-aged controls (Figure 3 and Figure 4).

## 4. Discussion

### 4.1. DMFT Analysis

There is an ongoing problem of how the total dental caries index should be calculated in the population with mixed dentition, where it is predominantly reported to be higher due to the longer exposure time of primary teeth [6,16,29]. An interesting A-DMFT index was proposed to enable comparative research regardless of age, but its use has not yet been widely documented in the literature and requires more extensive research [30]. Some authors calculated the sum of dmft and DMFT or average score between dmf and DMF for patients with mixed dentition [1,31]. This method of calculation, although sufficient when comparing research groups, may not reflect the actual oral health status and exposure to caries. Abbass et al. used the deft index, in which “d” refers to decayed tooth indicated for filling, “e”—decayed tooth indicated for extraction, and “f”—filled tooth [16]. Sometimes, dmft and DMFT were evaluated separately for all study participants with a wide age range or dmft/DMFT calculation in relation to the age remained unclear [5,6,10,11,23,29,32,33]. Some authors decided to divide the study groups into age-dependent subgroups and either dmft or DMFT were estimated and compared separately [12,34,35]. Due to the different exposure time and varying dentition (primary, mixed, and permanent) the authors of the current paper have also created three age groups enabling a more reliable comparative analysis between cancer survivors and healthy controls. Dmft, dmfs, DMFT, and DMFS were determined for primary and permanent teeth, respectively. Whereas in order to establish the relationship between the level of caries and other factors not related to cancer, the sum of dmft and DMFT (dmft + DMFT) was calculated for each age group.

A higher incidence of caries in cancer survivors has been noted in many previous reports [8,11,12,15,21,23,34,35,36,37]. Lauritano et al. have published the outcome of a DMFT investigation in 52 leukemia survivors aged from 8 to 15 years who had almost twice the index compared to controls. No other variables were taken into consideration. The authors comprehensively described the possible causes of this phenomenon, but no survey has been conducted on this issue [8]. Some authors reported a significantly higher incidence of caries in cancer survivors compared to controls, but no age-dependent division was applied to the study groups and the dmft/DMFT index referred to the total study population with varying exposure time [11,15,23,36]. In one study, a comparison between individuals younger and older than 5 years at cancer diagnosis was made, resulting in higher dmft in the younger and significantly higher DMFT in the older group, no matter how old the patient was at the dental examination [11]. Defabianis et al. also examined young patients being in remission of cancer disease and dmft/DMFT was evaluated in relation to the type of cancer and the age at diagnosis. Individuals treated before 5 years of age presented with lower mean DMFT regardless of treatment protocol. However, the remission time was also not taken into account and the study design did not include a control group [32]. Dens et al. also formed a division based on the age at cancer diagnosis (0–5 and 6–10) to compare FT/ft, FS/fs, DT/dt, and DS/ds scores in both cancer and control groups. Predominantly more teeth with active caries were observed in cancer survivors with a significant difference in relation to permanent dentition in the “older” group (DT, DS). Although healthy children in the study were matched in terms of age, race, and social class, the outcome may not reflect the actual situation because remission time was not revealed in the paper [12]. An interesting comparison was made among survivors who experienced different cancers. The highest dmft values were found in neuroblastoma survivors and the highest DMFT scores were observed in patients suffering from Hodgkin’s lymphoma. It is likely that survivors were examined shortly after anticancer therapy completion because neuroblastoma is known to be diagnosed at a very young age, unlike lymphomas being treated predominantly in adolescents. However, detailed data on remission time were not disclosed in the study [9]. Similar observations were made in another study, in which a higher prevalence of dental caries in younger study survivors was associated with a short time after anticancer therapy completion [1]. Long remission period may have a significant impact on caries expression and comparative analysis of groups formed by the age at cancer diagnosis may lead to false conclusions. It is more likely that the patient’s age at dental examination and remission period are more decisive. Following from this, the factors such as oral hygiene level or socioeconomic status seem to be more risky when it comes to caries expression, regardless of patient’s medical history. According to one cited study conducted on healthy children, caries prevalence varied with age, being highest in children aged 8–9, followed by 10–12, and the lowest in participants at the age of 6–7 with no association found with the parental oral health status [33]. In turn, children aged 9–11 years had the highest deft/DMFT scores in the studied group ranged 6–15 years [6]. It is difficult to find analyses of caries incidence with age inclusion criteria similar to the present study. Therefore, the outcomes vary greatly not only due to differences resulting from ethnic inequalities. Some authors have found a positive correlation between dmft/DMFT scores and age either in childhood cancer survivors or controls, but dmft was not determined in participants older than 9 years [12,35]. The authors of the current paper have noticed that the mean DMFT increased with age, while mean dmft was lower in subsequent age subgroups in the total study population ranged 5/8–18/0 (Table 2a–c, Figure 2). This depends on the replacement of teeth at a young age, which is typical of mammals. Similar results were previously noted in the literature either in cancer survivors or in healthy participants [16,35,38]. Regarding the study of Dens et al., apart from the 6–9 age group, cancer survivors presented with higher dmft/DMFT and dmfs/DMFS scores with significant differences noticed only in the 14–17 age group, but remission time was not disclosed in the study [12]. In another study, the indices only for permanent teeth were determined for three age groups ranged 5–11, 12–15, and 16–25 years and DM scores were higher in all survivors with a significant difference noticed in two last groups. The differences between the mean values and level of significance were increasing with age [10]. When it comes to our research, although the overwhelming number of mean dmft/DMFT, dmfs/DMFS, and dmft(s) + DMTF(S) scores in survivors were higher than in the controls, no statistically significant differences between the study groups have been found. An exceptionally lower mean DMFT and DMFS in the middle-aged survivors are even more surprising because a relatively larger number of patients in this group no longer had primary dentition, and therefore might have had a higher dental age. However, the median and DMFT range in both groups were the same. It is worth mentioning that all the groups of survivors presented with a very long mean remission period: 50.83, 77.0 and 75.14 months, respectively (Table 2a–c). No statistically significant differences in dmft/DMFT scores were also reported in another paper, although an average time of 2 years and 2 months (range: 1–5 years) from anticancer therapy completion was very short. However, the influence of the age factor on the study’s results cannot be ruled out, because the mean age of the control group was 2 years higher than in survivors [5]. Oguz et al. studied non-Hodgkin’s lymphoma survivors with a very short remission time (mean: 2.6 year; range: 1–6.2 years), and the dmft(s) and DMFT(S) indices were higher compared to controls, but the differences were not significant [37]. Anticancer therapy did not significantly contribute to caries occurrence, also according to other research [25,39,40]. Regardless of the predominantly higher mean dmft(s) and DMFT(S) scores noticed in survivors in the current study, the prevalence of dental caries in the entire tested group was found to be 5% lower than in controls (Figure 1). This value, also reflecting the situation in the examined group, is not often discussed in the literature.

Probably, the relatively small number of patients undergoing head and neck radiotherapy did not influence the study outcome. Of four patients receiving head radiotherapy, only one female presented with a dmft + DMFT score higher than the mean of her age group. Even if this female had the shortest remission time, she was diagnosed with brain cancer, so radiotherapy might have been less risky. Up to 90% of the unstimulated saliva has its origin from sub-mandibular glands [41]. This is consistent with observations of the other authors who found caries experience in both CT and RT recipients to be similar to DMFT scores in patients receiving CT alone [4,11,36]. Although there are some literature reports about higher caries experience after post-radiation salivary gland damage, a lower salivary secretion rate is described to be dose-dependent and may be transient [1,15,23,39,42].

The studied survivors remained in long-term remission for an average of 65 months; therefore, other influencing factors could certainly take over the history of cancer. The pathogenesis of dental caries is usually believed to be associated with compromised oral hygiene, improper dietary habits, and unfavorable socioeconomic status of the family [19]. Some authors have also reported gender inequalities indicating that males seem to ignore their oral hygiene habits and are more likely to experience different oral diseases [11,14]. Contrary to men, women have a positive attitude towards maintaining proper oral health [14]. Paradoxically, women develop more dental caries. They exhibit a lower salivary flow rate and lower concentration of IgA. Earlier exposure on the oral environment because of early teeth eruption, hormone activity, and possible deficiency of X-linked amelogenin gene expression are supposed to be important risk factors [43]. This was also confirmed in the current study, with the significant differences within the youngest controls (Figure 4). Abbass et al. have observed the highest mean dmft/DMFT in male children for primary and permanent dentition and no correlation between deft and gender in patients with mixed dentition [16]. Guagnano et al. reported a significantly higher DMFT value in male survivors compared to females [11]. No gender-related inequalities can also be found in the literature [13,38]. The two older groups of the current studied survivors demonstrate a significant numerical predominance of females, but, in the middle-aged group, the caries incidence in permanent dentition is even lower than in the controls, with an equal gender distribution (Table 2a–c; Figure 3 and Figure 4). Due to conflicting reports and unequal gender distribution, the authors decided to omit this factor when assessing correlations in this study.

Anticancer therapy and following obligatory regular check-ups were reported as a reason for negligence in other areas of studied survivors’ lives and general health including oral hygiene and dental follow-ups. This is consistent with the current study result both in terms of the higher dmft/DMFT scores in survivors, as well as in terms of the f/F value, which was significantly higher in all the groups of controls (Table 2a–c). This is also likely due to the fact that the control group included patients who voluntarily attended a dental clinic. In contrast, a significantly lower DMFT score was diagnosed in transplant patients compared to non-transplant survivors. Relatively high F scores revealed in transplant recipients, resulting from the need to remain infection-free, indicates a common trend of caregivers directing their attention to the most important health problems at the moment. However, contrary to our findings, no significant differences were noticed between cancer survivors and controls in this study, and the F score significantly correlated with age only in the control group [10]. In another study, only cancer survivors were tested in terms of treatment results, and a higher FT score relative to DMFT was noted in children younger than 5 years at cancer diagnosis. Even if these results are not applicable to our study, the FT level exceeded the half DMFT score which did not happen in any of our groups, either survivors or controls [32].

Due to the non-statistically significant differences in caries experience between the study groups and long remission time, the authors found it necessary to investigate the relationship between the caries incidence and various factors affecting oral health.

### 4.2. Oral Hygiene: PI and Tooth Brushing Frequency

Plaque and gingival indices in cancer survivors were previously reported to be significantly higher than in controls, even though sometimes dmft/DMFT did not significantly differ between the study groups [5,21,36]. In our research, PI was higher in all the age groups of survivors compared to the healthy controls, but a significant difference was found only in participants ranged 5/8–9 (Table 2a). In this group, survivors with fair oral hygiene had a higher dmft + DMFT compared to survivors presented with a good one (*p* = 0.068) (Table 3a). In another study only PI in the test group was significantly higher, while no statistically significant differences were noted regarding gingival status and caries experience [37]. Similar to our results, no differences can also be found in gingival status between survivors and generally healthy population in the literature [9,35]. Oral health status in cancer survivors (expressed by PI and GI) comparable to that of healthy individuals in the current study may result from the fact that caregivers who declared problems with oral hygiene during CT confirmed that they were transient and short-lived. Most respondents were very aware of the need to maintain good oral hygiene. Twelve out of forty survivors (30%) declared problems with proper oral hygiene during CT. Nine of them have experienced transient oral complications during hospitalization. On the contrary, fifteen study survivors who developed oral complications did not report any problems with oral hygiene. The most frequent mucositis is diagnosed in less than 5% of CT recipients as mild to moderate starting 7–10 days after toxic drugs’ administration and resolving within 1 to 2 weeks [7]. In our study, problems with maintaining proper oral hygiene during CT did not affect dmft + DMFT level (Table 3a–c). However, these relationships should not be analyzed due to the fact that tested values are not temporally related due to the long remission time. The assessment of the relationship between caries status and PI score taken during dental examination seems to be more reliable. Although the mean PI in the youngest survivors’ group was found to be higher than in the controls (*p* = 0.055), this was a moderate score compared to the results in the entire study. Moreover, although the difference was not significant, the youngest controls were diagnosed with a higher mean GI score (Table 2a–c). The level of hygiene in this age group strongly depends on caregivers; therefore, despite the shortest mean remission time shown in the youngest survivors, the factors relating to parental involvement should be taken into account. Notwithstanding the above, an increased dental plaque level has been identified as a risk factor for caries occurrence [15,44]. This was confirmed in our research, because as a result of the evaluation of the relationships between PI and dmf + DMFT, a strong positive correlation was revealed in both younger groups of survivors (Table 3a,b).

Numerous responsibilities related to monitoring the survivors’ condition after anticancer therapy completion may distract attention from their other needs [1,10]. However, the authors did not investigate the regular dental visits as a risk factor for caries because individuals from the control group were randomly recruited from patients attending our outpatient’s dental clinic. A very common question about the age at the first dental visit was also included in the study survey, but caregivers had the greatest difficulty answering this issue [15]. Tooth brushing frequency is often recognized in the literature as an important contributing factor in maintaining proper oral health status [13,21]. A brushing frequency of more than once daily is reported to be associated with lower DMF values [15,45]. The authors of the current paper observed that 22.5% of the survivors and 31.25% of the controls brush their teeth at most once a day. However, no significant differences in dmft + DMFT scores between groups differing in terms of brushing frequency were revealed (Table 3a–c and Table 4a–c). A moderate negative relationship was noticed only in survivors ranged 5/8–9/0 (rho = −0.45, *p* = 0.063) (Table 3a). Various findings were shown between caries increment with oral hygiene practices so far [44]. In the study of Abbass et al. on healthy Egyptian children, dmft increased with decreased brushing frequency of primary teeth, and the deft index for mixed dentition was negatively correlated with the brushing frequency of the parent to the child and brushing frequency of the child. The highest mean DMFT was for children who brush their permanent teeth once a day maximum; however, no relationship was revealed [16]. Although the quality of brushing seems to overtake its frequency, there are study participants with high PI and DMFT values who declare brushing frequency at least twice a day. According to Proc et al., this fact should be related to unreliability of the respondents, because no relationship was found between declared brushing frequency and PI scores in their study [15].

### 4.3. Socioeconomic Status: Education Level, Employment, and Number of Siblings

Children’s oral hygiene habits and regular check-ups at the dentist depend on parental involvement [6]. This in turn relates to their education, economic status, and time spent at work. Education level and income are usually taken into consideration when measuring socioeconomic status [17,46]. According to many analyses, socioeconomic status positively correlates with general and oral health [16,17,18,19,20,44,47]. Education in our country, more than household income, seems to be strong decisive factor for the condition of the oral cavity because of unlimited access to free health care. Individuals participating in the current study were recruited from children attending a clinic providing dental treatment under the National Health Fund. Moreover, income-related inequalities tend to increase with age without affecting the children’s group [48]. This is likely due to parents prioritizing their children’s health rather than their own, regardless of their financial status. For the same reason, the authors did not expect gender inequalities so often noted in adults [14]. Therefore, the inclusion criteria did not refer to gender. Moreover, when analyzing caries indices in the middle-aged group of survivors, dmft was higher, but DMFT was lower than in the controls, although the gender distribution was in favor of females (Table 2b; Figure 2 and Figure 3). The result is even more surprising because almost half of the survivors no longer had primary teeth compared to the control group in which almost all patients still had mixed dentition. Moreover, it would be even more difficult to establish any relationships if an additional analysis of gender distribution of dmft + DMFT in each study group was undertaken (Figure 4).

Education level is often analyzed in terms of oral health dependencies [15,44,47]. A negative correlation between DMF and education of caregivers has been widely documented [19,45,47]. The present study revealed a significant negative relationship only among controls aged 9/1–12/0, when the higher education was taken into account for both caregivers. A much higher mean dmft + DMFT was noted in almost all the study groups with elementary parental education compared to the S and PS level. Among groups with S and PS education, the mean dental caries scores were comparable (results not included in the paper). Proc et al. evaluated education level separately for mothers and fathers, and lower DMF scores were found in survivors whose parents had a higher education [15]. Based on the current study, after the evaluation was made separately for each parent, significantly lower mean dmft + DMFT values were found in middle-aged groups for higher educated fathers of survivors (*p* = 0.062) and mothers of healthy participants (*p* = 0.014). Higher education of mothers significantly negatively correlated with caries experience either in survivors or in controls ranged 9/1–12/0 (Table 3b and Table 4b). The significant relationship between a mother and a child in relation to oral health was earlier discussed in the scientific literature [46]. The study analysis also revealed a significant negative correlation between caries incidence and father’s education in survivors aged more than 9 years (Table 3b,c). Outstanding in evaluation groups ranged 9/1–12/0 is that they have similar characteristics in terms of the remaining socioeconomic variables analyzed in this study: the prevalence of children with both parents working and with at least two siblings. However, in terms of education, there are significant differences, i.e., almost twice as many mothers of survivors and almost five times as many fathers of survivors have PS school completed compared to the parents of controls. Parental education, of socioeconomic factors, may likely play a more significant role in dental caries occurrence, as only this age group of survivors have been diagnosed with lower dmft + DMFT index compared to the controls (Table 2b).

Education status influences a better social level of the family, what in turn may improve parental awareness of oral health habits and needs [15]. Associated with the level of education, parental employment may increase the economic position of the family and usually positively contributes to the dental condition [47]. Single parent families presented with higher rates of early childhood caries [18]. On the contrary, working caregivers as well as the higher number of children in one household may negatively correlate with meeting needs of the family members. Therefore, despite the employment of both parents, higher education, declared proper teeth brushing, and good diet, DMFT and PI values were found to be high in some study participants. Probably in the same context, the mother’s working status was analyzed in Qatari children aged 4–8 years, but no relationship with dental caries was noted [45]. In the current study, four out of six groups were observed to have a higher mean dmft + DMFT in families with only one working parent. Two remaining groups of survivors presented with higher rates of caries incidence, even if both parents were employed (results not included in the study tables). Parental employment moderately correlated with caries dmft + DMFT values in the oldest study groups and middle-aged controls. Correlations predominantly tended to be negative, and therefore could indicate that parental employment is associated with family income rather than time spent on child care (Table 3a–c and Table 4a–c). Nevertheless, the contradictory results should be associated with poor statistical power regarding the limited sample size.

Although the relationship between children’s oral health and the number of siblings is poorly documented, it is likely that an increase in the number of children may lead to a reduction in the quality of life of family members. Some previous studies documented a negative association in terms of this relationship [18,46,49,50,51]. It is more important whether the child is firstborn or not because it seems that an only child or oldest sibling is not concerned with the quantity–quality trade-off perspective. However, birth order is only just beginning to be discussed in the literature and its impact on oral health status is not clearly documented [19,31]. The authors of the current study considered more than one sibling to be a significant risk factor of caries incidence after they noticed that half of the control group had one sibling maximum. But, only among both younger control groups, dmft + DMFT scores were observed to be significantly lower in the study participants having at most one sibling (*p* = 0.067; *p* = 0.061) (Table 4a,b). However, a higher number of siblings tended to correlate with higher caries incidence in all the age groups (Table 3a,c and Table 4b,c). If the presence of at least one sibling had been considered an unfavorable factor compromising oral health care, it is more likely that the study results would have shown this relationship. An only child is described in the literature to present with better oral health status [46]. To sum up, parental employment and a larger number of siblings may be a negative factor in the context of caries incidence due to numerous responsibilities related to patients in remission of cancer.

### 4.4. Dietary Habits

A high-calorie diet is proven to strongly interfere with maintaining a caries-free dental status, irrespective of general health [16,19]. When it comes to CT recipients, they are more likely to intake cariogenic food due to the loss of appetite and taste alterations which may occur up to 14 weeks after treatment completion [5,7,10]. However, the majority of the current studied survivors had experienced a loss of appetite with an accompanying preference for non-cariogenic foods, which was also reported in the literature. Moreover, caregivers made great efforts to maintain proper oral hygiene despite difficulties. Therefore, due to a relatively long period of remission, current dietary practices are likely to contribute more strongly to the examined values. Only 29.8% of the school children declared not consuming a sugary diet, and 41% reported drinking carbonated beverages [6]. In the most recent study, children aged 4–8 years were surveyed about the number of daily servings of sweet food and drinks, with 1–8 and 8–13 recognized as low and intermediate, respectively. Caries experience was reported as lower in children with a low and intermediate number of servings of both sugary food and beverages [45]. A higher consumption of high-fiber grain products and lower snacking frequency were associated with lower caries experience [52]. Proc et al. did not notice any relationship between the frequency of eating sweets and caries indices [15]. Conflicting observations regarding the influence of dietary habits on caries incidence have appeared so far due to the low evidence of published research [44]. This is in agreement with our opinion that just as it is difficult for respondents to provide accurate information about their diet, it is even more difficult for researchers to classify these results in a reliable way. The current study criteria for qualifying for a risk group may seem excessive, even more so that snacking is considered helpful with meeting daily nutritional requirements. However, sugar consumption up to a maximum of 5% of daily energy intake is currently recommended in terms of the prophylaxis of both obesity and dental caries [53]. Moreover, many other accompanying factors contribute to the influence of diet on caries occurrence. The entire study population was divided into homogenous groups in terms of everyday lifestyle. The oldest groups ranged 12/1–18/0 presented with the highest prevalence in relation to the consumption of cariogenic food and drinks (71.43% of survivors; 60.00% of controls) and the highest dmft + DMFT scores (12.86—survivors; 11.53—controls). No differences and no dependencies related to the cariogenic diet were found either in the oldest or middle-aged study groups (Table 3b,c and Table 4b,c). Whereas, statistically significantly higher dmft + DMFT values were noticed in the youngest survivors and controls declaring a high-calorie food intake (*p* = 0.006; *p* = 0.038). The relationships in this matter were found to be strongly positive (rho = 0.67, *p* = 0.002; rho = 0.35, *p* = 0.036) (Table 3a and Table 4a). Therefore, an observation emerges that in the youngest survivors presenting with the shortest remission time, the factor of a cariogenic diet may accumulate with problems resulting from anticancer treatment. Attention to the above would be clinically of great importance.

### 4.5. Pregnancy Complications

In 20.00% of the survivors and 18.75% of the controls, mothers declared a complicated pregnancy, with the most frequently reported in the literature being PTB defined as a birth before 37th week of gestation, LBW as weight not more than 2500 g, and small-for-gestational age (SGA) as birthweight not exceeding the 10th percentile of expected for gestational age. Enamel opacities, immune system deficiency resulting in early colonization of the oral cavity by bacteria, and immature oral motor skills leading to early bottle-feeding are thought to be long-term adverse effects of compromised pregnancy and birth problems [54]. Nirunsittirat et al. have found an inverse association between PTB and dental caries in 3–4-year old children, but LBW and SGA were not related [54]. Numerous cohort studies were conducted under the influence of previous reports on the positive correlations between adverse pregnancy outcomes and dental caries [55]. The described relationships have not been confirmed [56,57,58]. The recent systematic reviews did not find the association of LBW and/or PTB with dental caries in primary dentition [59,60]. Our findings are consistent with these conclusions (Table 3a–c and Table 4a–c). Although the mean dmft was higher in each age group of cancer survivors, no statistically significant differences were established in the study (Table 2a–c). In view of such a long remission time, it would be difficult to find an explanation for the relationship between the variables in question.

## 5. Limitations

Significant heterogeneity in groups of survivors and various research methodologies in the literature made a comparative analysis difficult for the authors of the present paper [1]. Because great efforts were directed to obtain a highly age-matched healthy population, assembling the individuals matched on gender proved impossible. Similar to many studies, the small sample size due to the parental involvement in monitoring long-term adverse anticancer therapy effects is always the problem [9]. Moreover, the following circumstances could have influenced the study outcome: cancer survivors were recruited from among patients attending an oncology clinic, while healthy individuals reported voluntarily to the dental clinic.

## 6. Conclusions

The study findings suggest that there is no correlation between chemotherapy and dental caries. No statistically significant differences in caries incidence between cancer survivors and healthy peers have been found. Although treatment-related symptoms during chemotherapy contribute to the creation of a cariogenic oral environment, they were observed to be transient and current socioeconomic and oral health status had a much stronger impact on the caries incidence in the long-term studied survivors. However, in order to improve the quality of life, children with cancer require careful dental care during and after antineoplastic therapy. Thanks to cooperation between hematologists, pediatricians, and dental hygienists, the detrimental impact of chemotherapy on the oral cavity can be avoided. A multi-center study would be beneficial in achieving an appropriate sample size with better homogeneity.

## Figures and Tables

**Table 1:** Baseline characteristics of the survivors’ group.

		Number of Survivors (%)
**Age at cancer diagnosis (years/months)**	0–5	34 (85.00)
	5/1–10	6 (15.00)
**Type of cancer diagnosis**	Solid tumors ^1^	31 (77.50)
	Hematological cancers ^2^	9 (22.50)
**Type of anticancer treatment**	Chemotherapy ^3^	40 (100.00)
	Surgery	29 (72.50)
	Radiotherapy	13 (32.50)
**Duration of chemotherapy (weeks)**	0–24	6 (15.00)
	25–50	14 (35.00)
	51–75	7 (17.50)
	76–100	6 (15.00)
	100–125	7 (17.50)
**Age at dental examination (years/months)**	5/8–9/0	18 (45.00)
	9/1–12/0	15 (37.50)
	12/1–18/0	7 (17.50)
**Gender**	Female	24 (60.00)
	Male	16 (40.00)
**Problems during chemotherapy**	Oral health ^4^	24 (60.00)
	Oral hygiene	12 (30.00)
	Cariogenic diet	4 (10.00)

^1^ Nephroblastoma, neuroblastoma, medulloblastoma, rhabdomyosarcoma, hepatoblastoma, anaplastic ependymoma, infantile fibrosarcoma, sarcoma granulocyticum, teratoma malignum, embryonal primitive neuroectodermal tumor (PNET)/Ewing sarcoma (ES), yolk sac tumor, clear cell sarcoma, retinoblastoma, astrocytoma pilocyticum; ^2^ acute lymphoblastic leukemia, Hodgkin lymphoma, myelomonocytic lymphoma; ^3^ CWS 2006 Non-RMSlike HRG; CWS 2006 RMS-like; CWS 2002 (HR); SIOP 2001; 2002 PPGL; PPGGL SIOP December 2001; Euro-Ewing 99 PPGGL; Protocol I, III, IV PPGGL; Protocol II, III, IV PLGM recommended by PPGGL; TGM 95 PPGGL (HR); SIOPEL 3 (SR); ALL IC-BFM 2002 (SR, IR); ALLREZ BFM 2002; and BFM Interim 2004 HRG; ^4^ oral candidiasis, mucositis, aphthae, herpes, changes in taste, gingival haemorrhage, vomiting.

## Data Availability

The original data presented in the study are included in the paper; further inquiries can be directed to the corresponding author.

## Abbreviations

The following abbreviations are used in this manuscript:

CT	Chemotherapy
RT	Radiotherapy
dmft/s	decayed, missing, filled tooth/surface (primary teeth)
DMFT/S	Decayed, Missing, Filled Tooth/Surface (permanent teeth)
ft/s	filled tooth/surface (primary teeth)
FT/S	Filled Tooth/Surface (permanent teeth)
PI	Plaque Index
GI	Gingival Index
E	Elementary (education)
V	Vocational (education)
S	Secondary (education)
PS	Postsecondary (education)
Rho	Spearman’s correlation coefficient
PTB	Preterm birth
LBW	Low birth weight
SGA	Small-for-gestational age
